# Nanocarbon-Based Flame Retardant Polymer Nanocomposites

**DOI:** 10.3390/molecules26154670

**Published:** 2021-08-02

**Authors:** Yuan Yang, José Luis Díaz Palencia, Na Wang, Yan Jiang, De-Yi Wang

**Affiliations:** 1 Liaoning Provincial Key Laboratory for Synthesis and Preparation of Special Functional Materials, Shenyang University of Chemical Technology, Shenyang 110142, China; 15940340159@163.com (Y.Y.); na_jiangyan@sina.com (Y.J.); 2 Escuela Politécnica Superior, Universidad Francisco de Vitoria, Ctra. Pozuelo-Majadahonda Km 1800, Pozuelo de Alarcón, 28223 Madrid, Spain; joseluis.diaz@ufv.es; 3 Shenyang Research Institute of Industrial Technology for Advanced Coating Materials, Shenyang 110142, China; 4 IMDEA Materials Institute, C/Eric Kandel, 2, Getafe, 28906 Madrid, Spain

**Keywords:** graphene, carbon nanotubes, fullerene, flame retardant

## Abstract

In recent years, nanocarbon materials have attracted the interest of researchers due to their excellent properties. Nanocarbon-based flame retardant polymer composites have enhanced thermal stability and mechanical properties compared with traditional flame retardant composites. In this article, the unique structural features of nanocarbon-based materials and their use in flame retardant polymeric materials are initially introduced. Afterwards, the flame retardant mechanism of nanocarbon materials is described. The main discussions include material components such as graphene, carbon nanotubes, fullerene (in preparing resins), elastomers, plastics, foams, fabrics, and film–matrix materials. Furthermore, the flame retardant properties of carbon nanomaterials and their modified products are summarized. Carbon nanomaterials not only play the role of a flame retardant in composites, but also play an important role in many aspects such as mechanical reinforcement. Finally, the opportunities and challenges for future development of carbon nanomaterials in flame-retardant polymeric materials are briefly discussed.

## 1. Introduction

The use of carbon materials has a long history. Many types of carbon materials have been produced, from cheap graphite to expensive diamond, as well as carbon black, which is commonly used in the rubber industry. The development history of nano-scale carbon materials is relatively short, but they have exhibited excellent properties to attract the research community. Whenever a new nanocarbon material is found, it introduces revolutionary changes to the field of materials science and technology. In 1985 [1], fullerenes were first discovered. Fullerene is a kind of 0-D nanocarbon material with free-radical trapping properties. In 1991 [2], a 1-D tubular carbon nanomaterial called carbon nanotubes was discovered. In 2004 [3], graphene, a 2-D layered nanomaterial of a single atom in thickness, was discovered and became relevant due to its excellent properties. All of these materials can be regarded as allotropes formed by a large number of carbon six-membered rings [4]. In addition, there are other nanocarbon materials, such as carbon black and expandable graphite. Both have been used to prepare nanocomposites with strong mechanical properties, increased thermal stability, thermal conductivity, and electrical conductivity and flame retardancy [5,6,7,8].

Polymer materials are widely used in various fields because of their excellent properties. As an aspect to highlight, they are basically prepared by polymerization of organic compounds, so they usually have high flammability, which increases the risk of fire during their lifetime. Consequently, most of the polymer materials need to add flame retardants during the preparation cycle. Most of the traditional flame retardants are required to be added in higher amounts to achieve optimal flame retardancy properties in the basic polymer. This fact increases the cost of the resulting polymer materials and also reduces their mechanical properties. In addition, halogenated flame retardants, typically employed due to their high flame retardant efficiency, introduce safety concerns, leading to their being catalogued as a prohibited product by the European Union. In accordance with this, the preparation of a novel flame-retardant material with high performance, a low load in dosing, increased safety, and decreased environmental impact has become an urgent problem. An exploration of nanocarbons, as the most optimal fit to cover the mentioned conditions, has led the research community to explore innovative ideas for the preparation of a new and efficient flame retardant.

**Figure 1:**
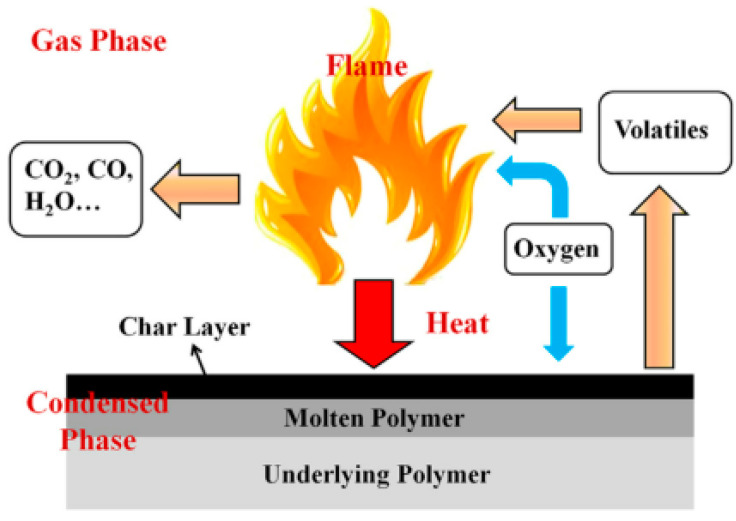
Illustration of a model of a flame retardant’s action [9].

Flame retardants in polymer materials mainly finish or block the combustion chain reaction in various ways. Generally speaking, the combustion process requires fuel, oxygen, and an energy source. Flame-retardant materials usually affect one or more of the mentioned three contributors. There exist two basic mechanisms to explain the flame retardant property, as illustrated in Figure 1. The first is the mechanism of the gas phase flame retardant that is related to the large number of active free radicals produced during polymer matrix combustion. These free radicals are necessary for the combustion chain reaction as well. Some flame retardants can capture free radicals, leading to the cutting off of the combustion chain reaction. Other flame retardants can produce inert gases, such as NH_3_, when they are decomposed. This fact permits us to dilute the oxygen and active free radicals, leading to a delay in the development of a flame. The second is the part of the flame retardant that can catalyze the formation of the carbon layer and strengthen it. The carbon layer formed on the substrate can block the contact of heat and oxygen with the underlying matrix material and reduce the diffusion of active free radicals so as to improve the flame retardancy. Most of the carbon nanomaterials can enhance the carbon layer and catalyze the formation of carbon, while others, such as fullerenes, also have the ability to capture free radicals [9].

With plenty of emerging studies on nano carbon materials in flame retardant materials, more and more nanosized carbon-based materials have been found to improve the flame retardancy of polymers. However, in this review, we mainly focus on the applications of graphene, carbon nanotubes, fullerene, expandable graphite, carbon black, nano carbon diamond, and their nanohybrids in flame-retardant polymer-based materials. Considering the application scope, nanocarbon materials are considered, among other things, for enhancing mechanical, thermal, and electrical properties. In relation to the manufacturing processes, insights are provided in relation to the preparation of multifunctional flame-retardant nanocomposites. Finally, the development opportunities and challenges of nanocarbon materials in the field of flame-retardant polymer materials are briefly reviewed.

## 2. Graphene

### 2.1. Synergistic Flame Retardancy of Graphene

Graphene has high thermal stability properties together with physical barrier functions. These characteristics make the graphene have flame retardancy properties to a certain extent. Pristine graphene cannot make a polymer matrix reach proper standard levels of flame retardancy [10]. Consequently, it is a common strategy to use graphene as a synergist in conventional flame retardant systems. We summarize the synergistic flame retardancy of graphene and its modified products in Table 1.

In recent studies, 2 wt% of graphene and 2 wt% of molybdenum disulfide (MoS_2_) were added to an ethylene vinyl acetate (EVA) matrix as a flame retardant synergist of aluminum hydroxide. As a consequence, the amount of aluminum hydroxide could be reduced to 36% to reach the UL-94 V-0 level. However, the EVA composite only added aluminum hydroxide and MoS_2_ can only reach the V-2 level. In this sense, graphene improves the dispersion of MoS_2_ in the matrix, leading to an enhancement in the flame retardancy properties [11]. According to the different amounts of graphene added in the intumescent flame retardant system, different mechanisms of action should be considered. After adding 0.5 wt% graphene, the peak heat release rate of a PP/IFR sample decreased by 73 kw/m^2^ compared with the sample without graphene. This result proved that 0.5 wt% graphene shows a better flame retardant synergistic effect. When increasing the amount of graphene, the flame retardant performance of the subject composite reduces because of the increasing viscosity of the polymer at melt that hinders the formation of the expanded carbon layer [12]. In another direction, montmorillonite (MMT) is a kind of inorganic nanomaterial with excellent thermal stability. It has attracted much attention as an inorganic flame retardant synergist. MMT tends to agglomerate in polymer matrices, which not only weakens the flame retardant effect, but also has a negative influence on the mechanical properties in the matrix material. The limiting oxygen index of composites prepared by polyimide aerogels with 5 wt% graphene, 10 wt% MMT, and 5 wt% graphene/10 wt% MMT was 46%, 45.4%, and 55%, respectively. The combination of graphene oxide (GO) and MMT showed excellent synergistic dispersion along the matrix, leading to a significant improvement compared with montmorillonite alone [13].

Graphene, as a synergist, can be used together with a variety of traditional flame retardant materials, including layered double hydroxide (LDH) and 9,10-dihydro-9-oxa-10-phosphaphenan-threne-10-oxide (DOPO). It is to be considered that LDH has difficulties forming homogeneous products with epoxy resin. The flame retardant property of the epoxy resin composite can be improved by adding graphene to ameliorate the dispersion of the layered double hydroxide. Through the synergistic dispersion of graphene and LDH, the viscosity at melt is increased to inhibit the spread of a flame during the matrix combustion process. The THR of the composite can be reduced from 33.4 KJ/m^2^ to 24.6 KJ/m^2^ by adding 2.5 wt% graphene and 2.5 wt% LDH, respectively. The THR value was lower than with the addition of GNS (27.8 KJ/m^2^) or LDH (25.7 KJ/m^2^) alone at the same addition level, 5 wt%. The results show that LDH and graphene have a better synergistic flame retardant effect. The peak heat release rate of LDH/DOPO/EP nanocomposites was reduced by 46% compared with that of neat EP at the loading of 2.5 wt% DOPO and 2.5 wt% graphene, respectively. The THR of ER, ER/GNS 5, ER/DOPO 5, and ER/GNS 2.5/DOPO 2.5 composites was 72.5 MJ/m^2^, 66.6 MJ/m^2^, 53.1 MJ/m^2^, and 48.1 MJ/m^2^, respectively. Therefore, DOPO and graphene have a synergistic flame retardant effect in epoxy resin systems [14]. Guan et al. [15] combined graphene nanosheets modified by a silane coupling agent (m-GNPs), spherical alumina, and magnesium hydroxide to prepare flame-retardant and thermally conductive epoxy resin composites. Achieving a high thermal conductivity requires us to add significant concentrations of alumina. This leads to an increase in the epoxy resin’s viscosity. Using 7% m-GNPs instead of the same amount of alumina can not only reduce the processing difficulty of epoxy resin, but also form a three-dimensional thermal conductivity framework with spherical alumina, which greatly improves the thermal conductivity. In the same direction, graphene and magnesium hydroxide can form a synergistic flame retardant as well. The epoxy resin composite with 68% alumina, 7% graphene nanosheets, and 5% magnesium hydroxide has a thermal conductivity of 2.2 W/(MK), a limiting oxygen index of 39%, and a V-0 rating of UL-94. In contrast, the epoxy composites with only spherical alumina failed in the UL-94 vertical burning test. The peak heat release rate decreased by 54% compared with neat EP, showing the excellent properties of graphene as a kind of nanocarbon material.

Nowadays, polylactic acid (PLA), as a kind of degradable polymer material, has become attractive because of its sustainability (mainly it avoids environmental deterioration and does not require many petroleum resources). However, polylactic acid is highly flammable and produces serious dropping during combustion. Improving the flammability requires us to add a phosphorus-based flame retardant into the PLA matrix in relatively large quantities at a sufficient level to reduce the mechanical properties. When 0.5 wt% graphene was added into a foamed polylactic acid composite containing a 15 wt% phosphorus flame retardant, the LOI increased to 29.2% and reached the V-0 level of UL-94. The LOI of PLA with a 15 wt% intumescent flame retardant is 27.9%. This proves the synergistic effect of a small amount of graphene in an intumescent flame retardant system. The addition of graphene not only improves the flame retardancy of PLA, but also reduces the effect of the intumescent flame retardant and maintains the cellular structure of the foamed PLA composite [16].

**Figure 2:**
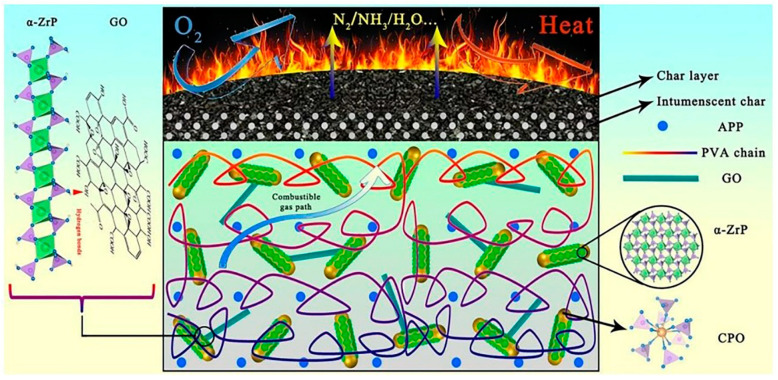
The possible flame retardant mechanism of ZCG/APP/PVA composites [17].

The flame retardant synergistic effect of original graphene is limited. This is an important drawback that typically produces difficulties with meeting the flame retardant demand in most application scenarios. Nonetheless and as previously pointed out, an improved performance flame retardant can be prepared by combining graphene with other materials. Leng et al. [17] prepared a α-zirconium phosphate (α-ZrP)/cerium phosphate (CPO)/graphene oxide (GO) nanocomposite (ZCG) flame retardant. As shown in Figure 2, α-ZRP links graphene oxide and cerium oxide through hydrogen bonds to form a synergistic effect, which improves the dispersion of the flame retardant. Compared with the composites with 10 wt% APP, the LOI of the composites increased from 28% to 36% after replacing the same amount of app with 5 wt% ZCG. At the same time, without adding graphene oxide, the limiting oxygen index of the composites is lower than 36%, which fully proves the synergistic effect of graphene on flame retardancy.

Through the condensation reaction of oxygen-containing functional groups on graphene oxide and amino groups on ammonium polyphosphate (APP), the final product was defined as I-G2, which is used for improving the flame retardancy of PBT. The addition of graphene enhances the carbonization performance of ammonium polyphosphate and improves the flame retardancy and mechanical properties. PBT/30 wt% IFR composites failed in the vertical combustion test. Surprisingly, PBT/20 wt% IFR/0.3 wt% I-G2 composites reached the V-0 rating of UL-94 tes. These results indicate that graphene oxide has obvious synergistic flame retardancy [18].

Bio-based polyphosphate (BPPT) is a new type of environmentally friendly phosphorus with promising flame retardant properties. Flame-retardant polylactic acid composites were prepared by using polyethyleneimine-modified graphene oxide (M-GO) as a synergist. When 2.4 wt% bio-based polyphosphate and 0.6 wt% M-GO were added, the LOI reached 36% and a UL-94 V-0 rating was obtained. Compared with the PLA composite with 3 wt% BPPT, the LOI of the composite with M-GO increased from 33.6% to 36.0%. The droplet behavior of the composites was also restrained in the vertical combustion experiment. If the loading amount of modified graphene is increased on this basis, the flame retardancy will deteriorate. This may be due to the fact that, in this flame retardant system, bio-based polyphosphate mainly plays the role of the gas phase flame retardant. If the added amount of graphene is too large, the free radical quenching performance of the bio-based polyphosphate will be affected. Furthermore, the addition of graphene permits us to enhance the mechanical properties and flame retardancy simultaneously [19].

**Figure 3:**
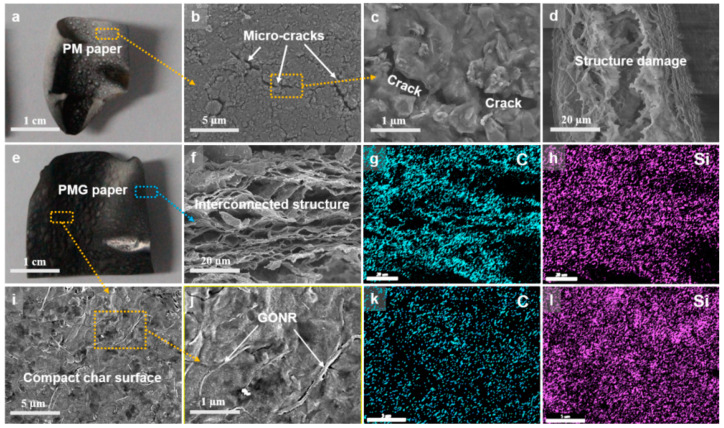
(**a**) Digital photograph and (**b**–**d**) surface/cross-section SEM images of P_1_M_2_ paper after the burning test, showing some cracks on the surface and obvious structural damage. (**e**) Digital photograph, (**f**–**h**) surface SEM and EDS mapping images, and (**i**–**l**) cross-section SEM and EDS mapping images of P_1_M_1_G_1_ paper after the burning test, confirming the formation of a stable interconnected structure and a compact char surface [20].

Yu et al. [20] prepared water-based graphene oxide nanoribbon/montmorillonite/polyethylene glycol hybrid (PMG) networks by a low-temperature, evaporation-induced assembly process. This kind of material can achieve a bionic design similar to nacre, and it can be made into PMG nanocomposite paper materials with high mechanical properties and flame retardancy. The results show that the material has no obvious curl deformation after combustion, and the morphology of the carbon residue has no cracks. A PMG layer of about 200 nm was prepared on the surface of a polyurethane foam by dip coating. Adding 10–30 wt% PMG material could make the composites achieve better flame retardancy and the peak heat release rate decreased by 46%. As seen in Figure 3, the carbon residue of the polyurethane foam coated with PMG material was increased greatly after a cone calorimetry test, and the structure of the polyurethane foam remained after combustion.

Transition metal elements have the ability of catalytic carbonization. It is very useful to combine a transition metal with graphene to form a highly efficient flame-retardant synergist that integrates catalytic carbonization and a physical barrier. Chen et al. [21] prepared an iron-graphene flame retardant synergist by adding iron powder and hydrochloric acid in a graphene oxide suspension, and used it in flame-retardant thermoplastic polyurethane materials with melamine pentaerythritol phosphate (MPP) as the flame retardant. The composite with only 0.25% iron–graphene (IG) obtained excellent flame retardancy with a limiting oxygen index of 32.75% and a UL-94 grade of V-0, and the peak heat release rate decreased from 2192.6 kW/m^2^ of pure TPU to 187.2 kW/m^2^. The limiting oxygen index (LOI) of the composites without IG was 30.5%, and these composites only reached the V-1 level in the vertical combustion test. SEM images of the carbon residue surface show that the carbon residue surface of the samples with the flame retardant was very smooth and compact, without cracks and holes. During combustion, the high-quality carbon layer can protect the underlying matrix material from barrier heat and combustible gas and achieves excellent flame retardant performance.

Natural fiber-reinforced polypropylene has excellent properties, but its high flammability limits its application. The question of how to enhance its flame retardancy is a very important subject. Although APP, magnesium hydroxide (MH), and other traditional flame retardants can provide better flame retardancy, their addition amount is usually large. By adding exfoliated graphene nanosheets as a flame retardant synergist, the flame retardant performance of traditional flame retardants can be improved. Graphene nanosheets achieve flame retardancy and a synergistic effect mainly through a physical barrier and the promotion of carbonization [22].

Ionic liquid is a kind of solvent and material in line with the concept of green chemistry. It has high thermal stability and low flammability. It is a compound with great potential to become an excellent flame retardant. Wan et al. [23] used the silane coupling agent KH550 and the ionic liquid 1-butyl-3-methylimidazole hexafluorophosphate (PF_6_-ILs) to modify the surface of graphene oxide (GO). By adding 0.1 wt% PF6, ILs@GO and 14.9 wt% MPP were found to have the best flame retardant properties. The results show that the total heat release (THR) and total smoke release (TSP) of the EP/MPP/PF6-ILs@GO composite decreased by 24.3% and 53.4%, respectively. In this study, the silane coupling agent was very important to the surface modification of graphene oxide. Nano carbon materials are usually easy to agglomerate. The agglomeration of graphene oxide can be inhibited by modifying it with a silane coupling agent, which improves the dispersion and compatibility of nano carbon materials in epoxy resin. This can improve the properties of flame retardant composites.

**Table 1:** Synergistic effect of graphene-based nanomaterials and other flame retardant additives on the flame retardant properties of polymer composites.

Polymer	Loading of Graphene Nanomaterials	Type and Loading of Other Flame Retardant Additives	Highlights	Ref
EVA	2 wt%	ATH 36 wt%, MoS_2_ 2 wt%	PHRR decreased from 1815 kW/m^2^ to 377 kW/m^2^	[11]
PP	0.5 wt%	IFR2 4.5 wt%	PHRR decreased from 1025 kW/m^2^ to 140 kW/m^2^	[12]
PI	5 wt%	MMT 10 wt%	UL-94: V-0 rating, LOI: 55%	[13]
EP	2.5 wt%	DOPO 2.5 wt%	PHRR reduced from 1194 kW/m^2^ to 396 kW/m^2^	[14]
EP	7 wt%	Al_2_O_3_ 68 wt%, MH 5 wt%,	UL-94: V-0 rating, LOI: 39%	[15]
PLA	0.5 wt%	phosphorus-containing flame retardant 15 wt%	UL-94: V-0 rating, LOI: 29.2%	[16]
PBT	0.3 wt%	IFR 20 wt%	UL-94: V-0 rating, LOI: 25.4%	[18]
TPU	0.25 wt%	MPP 14.75 wt%	PHRR decreased from 2192.6 kW/m^2^ to 187.2 kW/m^2^	[21]

### 2.2. Inorganic Hybrid Graphene

**Figure 4:**
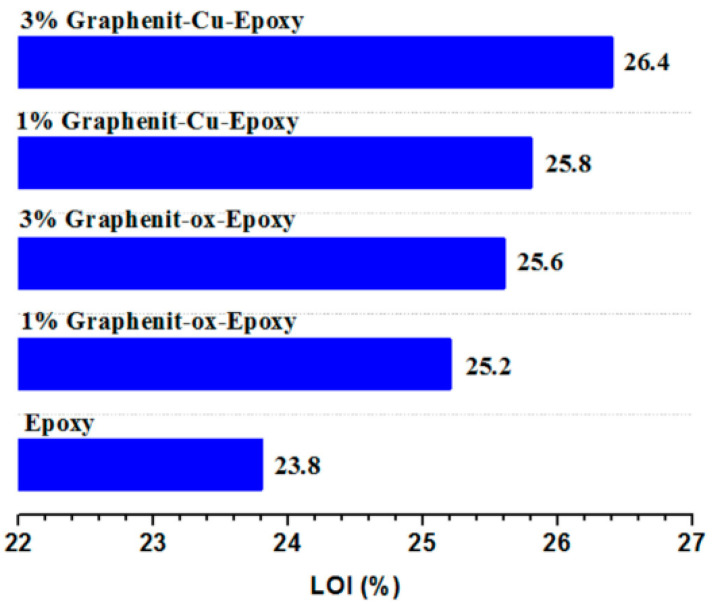
LOI of epoxy resin composites modified by copper-doped graphene [24].

Graphene is usually used as a flame retardant synergist in composites, but it is difficult to use it as a flame retardant alone. However, based on its excellent properties, it would be useful to prepare a highly efficient flame retardant by the modification of graphene. Liu et al. [24] successfully prepared copper-doped graphene and applied it to a flame retardant epoxy resin. According to Figure 4, after adding 3 wt% copper-doped graphene, the LOI of the EP composites increased to 26.4% compared with neat EP. On the other hand, the peak heat release rate of the composites decreased by about 34%, and the smoke suppression performance also improved. In addition, the thermomechanical properties of the composites also changed.

Reduced graphene oxide/aluminum hypophosphite (AHP/RGO) hybrid materials were prepared by a one-pot method to improve the flame retardancy of aluminum hypophosphite. The cone calorimetric test results of a PBT composite with 20% AHP/RGO show that the PBT/20 wt% AHP/RGO composites can not only reduce the heat release rate but also reduce the release of CO and toxic gas when the material is burned compared with the addition of aluminum hypophosphite alone [25].

In another direction, black phosphorene (BP) has a unique two-dimensional structure and is a phosphorus-containing compound that has the potential to become a highly efficient flame retardant. However, the mechanical properties of the polymer will deteriorate when black phosphorene is added to the polymer matrix. Ren et al. [26] added a mixture of black phosphorene and graphene into a high-pressure nano homogenizer to prepare black phosphorene/graphene/waterborne polyurethane (WPU) composite materials. It was shown that a phosphorus–carbon bond formed between the edge of the black phosphorene nanosheet and the graphene nanosheet. The results show not only an improvement in the flame retardancy of the composite materials, but also a 7-fold increase in the Young’s modulus of the BP/G/WPU compared with the BP/WPU.

The flame retardancy of fabrics and fiber materials has become an important research topic with interesting implications. Most flame retardants added to the fiber will affect the fiber’s color and reduce the mechanical properties. Xiao et al. [27] prepared nanofiber composites with red phosphorus hybrid graphene as the core and nylon as the shell by electrospinning. In this way, the flame retardant composite fiber can maintain its color and improve the flame retardant and mechanical properties. When red phosphorus is burned, it produces phosphorous compounds to promote the formation of carbon, while graphene can strengthen the carbon layer and prevent combustion, forming a synergistic flame retardant effect.

**Figure 5:**
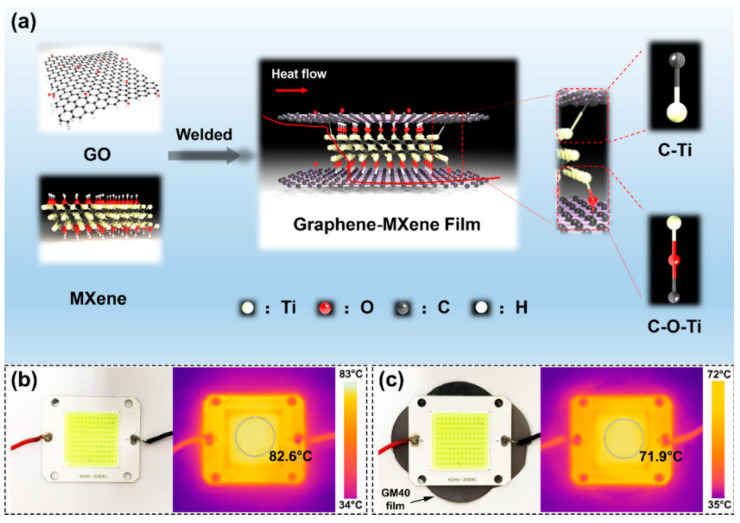
(**a**) Combination mode of MXene and graphene (**b**) Optical image of the LED and the corresponding IR thermal image. (**c**) Application of the GM paper as an ultra-thin heat sink for LED and the corresponding IR thermal image [28].

MXene is a two-dimensional transition metal carbide, nitride, or nitrogen carbide. MXene has two-dimensional lamellar structure similar to that of graphene and high thermal conductivity. Consequently, it is considered to be a material with promising thermal conductivity and flame retardant properties. Liu et al. [28] prepared MXene/graphene nanocomposite films by reduction and thermal welding at a suitable temperature. Figure 5a shows a model of the interaction between MXene and graphene. Figure 5b,c indicate the interesting performances of the MXene/graphene nanocomposite as a heat dissipation material for electronic components. According to the test results on a micro-combustion calorimeter, the peak heat release rate is only 10 wg^−1^. Indeed, this indicates that the MXene/graphene nanocomposite film is an excellent flame retardant.

Layered double hydroxides are a kind of nano filler that have a high aspect ratio. The natural agglomeration phenomenon requires us to study the surface modification of layered double hydroxides to improve their dispersion. Edenharter et al. [29] modified the surface of layered double hydroxides and graphene oxide at the same time in order to prepare PS/LDH-DBP-5 wt%/GO-DDA-0.5 wt% composites. The LOI did not change significantly, but the peak heat release rate of the composites decreased by 47% compared with neat PS. Moreover, the combustion time was prolonged.

Mesoporous zinc ferrate (MZF) is a material with high flame retardant potential because of its porous structure and the fact that it contains iron and zinc. Yang et al. [30] synthesized a mesoporous zinc ferrate hybrid graphene oxide (MZF-GO) composite by a hydrothermal method. By combining the catalytic performance of mesoporous zinc ferrate with the physical barrier function of graphene, improved flame retardancy behavior was obtained. When the addition amount of MZF-GO was 3 wt%, the peak heat release rate of the composite was reduced by 39%. This fact provides a new perspective for the application of an efficient environmental flame retardant.

**Figure 6:**
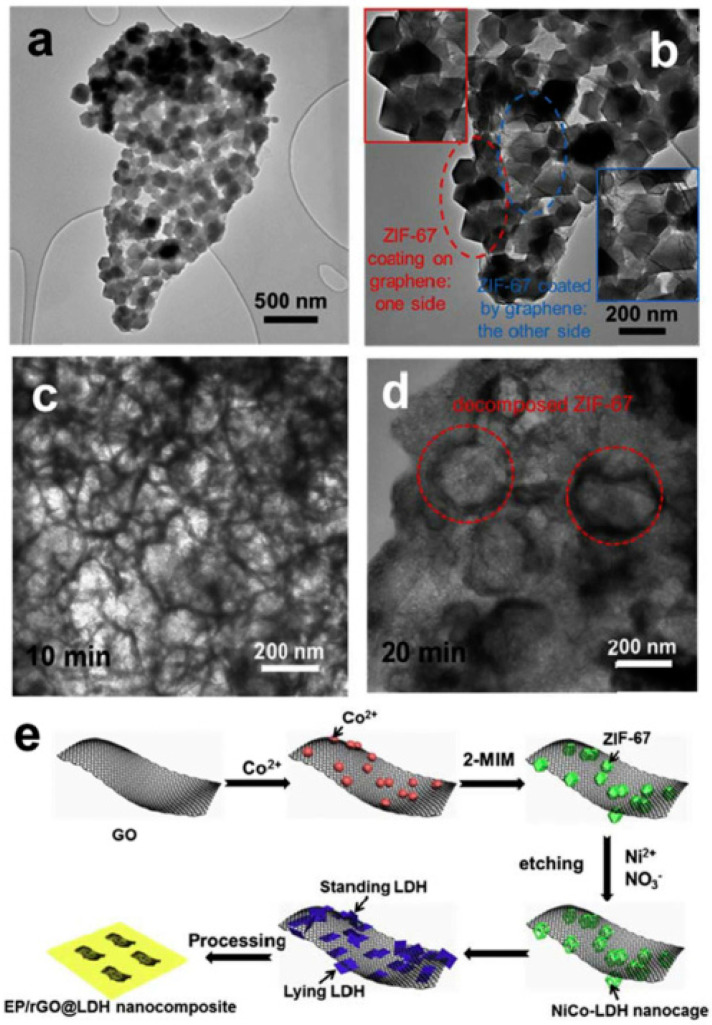
(**a**) TEM image of rGO@ZIF-67; (**b**) High-magnification TEM image of rGO@ZIF-67; (**c**,**d**) TEM images of the as-synthesized products obtained at different time intervals (10 min (**c**), 20 min (**d**)); (**e**) Schematic illustration of the synthesis of rGO@LDH and the preparation of EP/rGO@LDH nanocomposites [31].

Pan et al. [31] developed a flame-retardant insulation epoxy resin composite that can rapidly dissipate heat. These properties come from the preparation of nickel–cobalt bimetallic layered hydroxides (NiCo-LDH). As shown in Figure 6, the acid-sensitive bimetallic organic frameworks are used as sacrificial precursors to form NiCo-LDH on graphene. According to the results of cone calorimetry, the pHRR of the EP/2%wt rGO@LDH composites was lower than that of EP/2 wt% LDH composites and neat EP. As pointed out above, graphene promotes heat transfer and catalytic carbonization, which is the main reason for the rapid heat dissipation and high flame retardancy properties.

Traditional intumescent flame retardants consist of an acid source, a gas source, and a carbon source. Chavali et al. [32] synthesized potassium carbonate hybrid graphene by a hydrothermal method to produce a highly efficient intumescent flame retardant. Note that graphene can be used as a carbon source, and it can catalyze the formation of carbon during combustion. In addition, potassium carbonate will release carbon dioxide as a foaming agent at high temperatures. This material was used to prepare a flame-retardant fabric.

Aluminum hydroxide (AH) is an important traditional flame retardant; nonetheless, it is added in large amounts (usually 50%), affecting greatly the mechanical properties of pristine materials. Jeon et al. [33] prepared heavy aluminum hybrid graphene nanosheets (AlGnP) by physical blending with aluminum content of more than 30%. Through a large number of characterization trials, a covalent bond between the aluminum and the graphene was proven to exist. The thin films were prepared by adding heavy aluminum graphene nanosheets to polyvinyl alcohol (the content of AlGnP was 25 wt%). The pure polyvinyl alcohol burned out after 12 s, while the modified polyvinyl alcohol did not burn under the same conditions, but had a certain amount of deformation. The results show that the heavy aluminum graphene nanoflakes have excellent flame retardancy properties.

**Figure 7:**
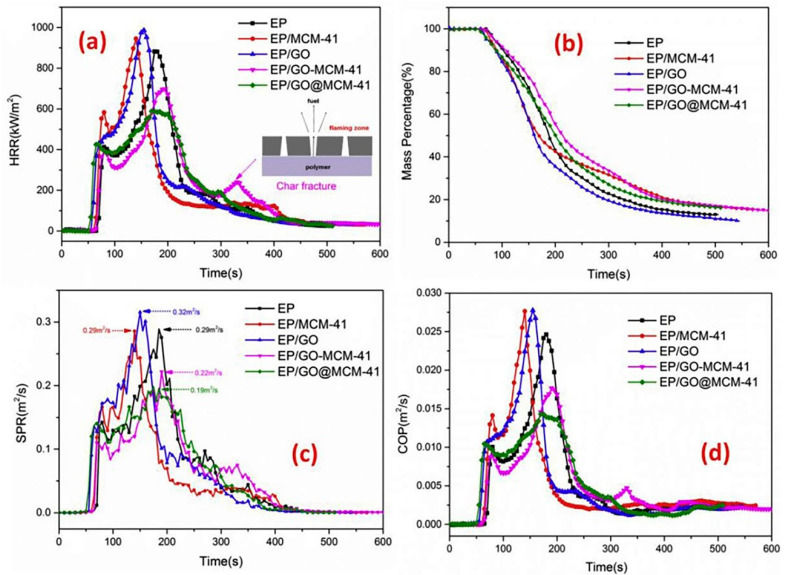
(**a**) HRR, (**b**) mass profiles, (**c**) SPR, and (**d**) COP of EP, EP/MCM-41, EP/GO, EP/GO-MCM-41, and EP/GO@MCM-41 [34].

In the same direction, a hierarchical nanohybrid (GO@MCM-41) was prepared by the covalent assembly of mesoporous MCM-41 nanospheres on graphene oxide (GO) nanosheets to improve the flame retardancy of epoxy resin (EP). Figure 7 shows the CCT results of the EP/GO@MCM-41 composites. The pHRR and SPR of the EP/GO@MCM-41 composites were remarkably reduced compared with neat EP. In this study, the content of all the flame-retardant additives was 2 wt%. The flame retardancy of the epoxy resin composites was enhanced and attributed to GO@MCM-41. The dispersion of MCM-41 was improved due to the existence of graphene nanoflakes, and synergistic catalytic carbonization was produced [34].

**Figure 8:**
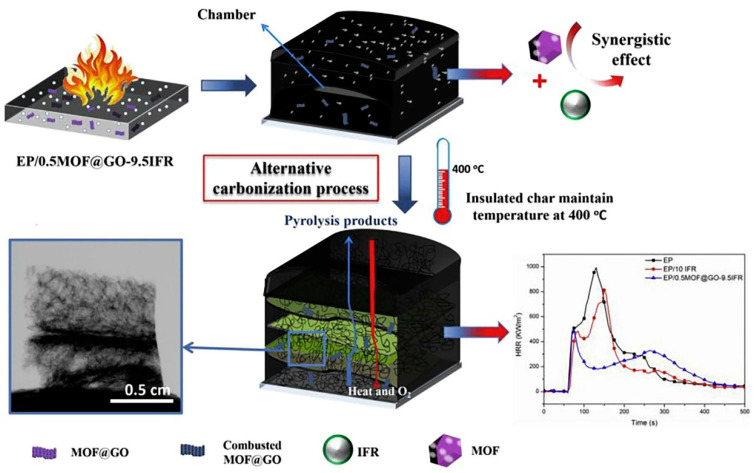
Proposed fire retardancy mechanism of intumescent EP with MOF@GO (EP/0.5MOF@GO-9.5IFR). (A colored version of this figure can be viewed online) [35].

Red phosphorus hybrid graphene nanocomposites were prepared by means of one-step ball milling, leading to a flame-retardant polyimide foam. The composite foam is characterized by a light weight, good mechanical properties, and strong flame retardancy. During the physical hybridization process, red phosphorus forms a phosphate group on the edge of the graphene and forms nanoparticles on the same graphene surface. This makes the red phosphorus hybrid graphene have good dispersion in the polymer matrix and high phosphorus content, which is the basis of its flame retardant properties [5]. Bimetallic metal–organic framework (MOF) and graphene oxide (GO) nano-hybrids (MOF@GO) have been used as the synergist in intumescent fire retardants (IFRs). According to Figure 8, these nano-hybrids have a novel alternative dynamic carbonization phenomenon in the accumulated char layer of epoxy resin (EP) composites, and the second heat release rate peak of the EP/0.5 wt%MOF@GO-9.5 wt% IFR was reduced by 59% compared with the EP/10 wt% IFR [35].

### 2.3. Layered Coating of Modified Grapheme

**Figure 9:**
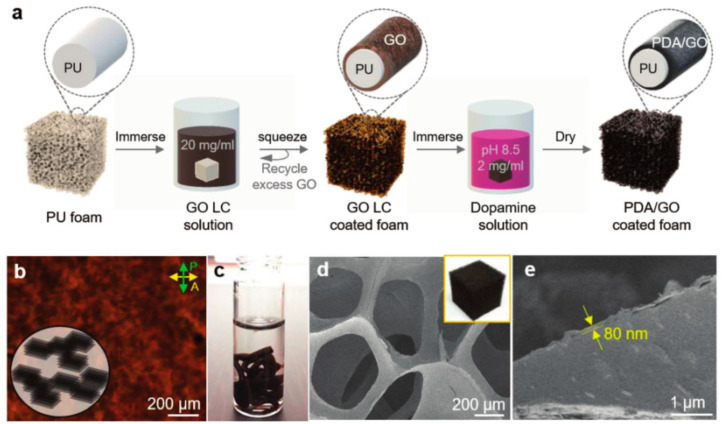
(**a**) Scheme for preparing the PDA/GO FR nanocoating on the PU foam assisted by the LC phase of an aqueous GO dispersion. (**b**) Polarized optical texture of a concentrated GO dispersion (20 mg mL^−1^). (**c**) Photograph of the GO LC after extrusion through a pipet into a dopamine solution (2 mg of dopamine mL^−1^ in Tris-HCl buffer, pH 8.5). (**d**) SEM image of dried (2.5/2.5 wt%) PDA/GO-coated PU foam. The inset is a photograph of the same foam. (**e**) Cross-sectional SEM image of the PDA/GO-coated PU foam [36].

Polyurethane foam is a material with excellent properties; nonetheless, its high flammability may induce limitations towards applications. In addition, many flame retardants will affect the foaming process of polyurethane or reduce the mechanical properties. Kim et al. [36] used an aqueous liquid crystalline (LC) graphene oxide (GO) scaffold and polydopamine to form a PDA/GO nanocoating on the surface of flexible polyurethane (PU) foam (Figure 9). The PDA/GO-coated PU foam showed significant flame retardant performance, reflected by a 65% reduction in the PHRR at a 5 wt% PDA/GO loading.

Polydopamine is particularly interesting as it has excellent adhesion and free-radical absorption properties. Using the layer-by-layer self-assembly procedure with the mentioned flame retardants, different coatings can be firmly bonded together and play the role of a gas phase flame retardant. The smoke release rate and the total smoke emissions of the polyurethane foam decreased by 50.2% and 58.7%, respectively, indicating that the smoke suppression performance of the polyurethane foam was outstanding [37].

**Figure 10:**
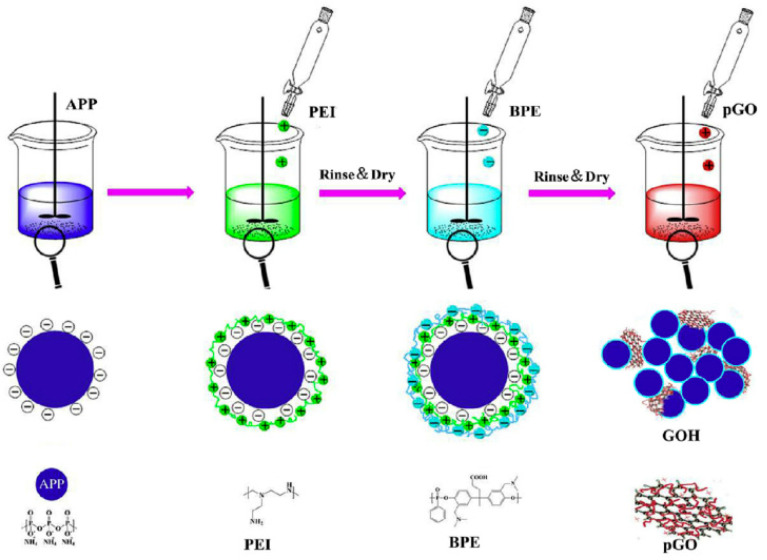
Schematic illustration of GOH fabrication via the LbL method [38].

Jing et al. [38] used organic solvent-free self-assembly to synthesize a bio-based flame retardant/graphene oxide hybrid (GOH). Figure 10 shows the mechanism of self-assembly by electrostatic interaction. This modification method improved polylactic acid composites with 15 wt% GOH to the V-0 level of vertical combustion, increased the tensile strength by 6 times, and increased the impact strength by 86.7%. Graphene is the basis of these effects. It provides an adhesion point for the bio-based flame retardant and improves the contact area between the flame retardant and the matrix due to its large specific surface area.

Tannic acid (TA) is a kind of biomass material that can be extracted from a variety of trees. It has a large number of phenolic hydroxyl groups in its structure, so it has a strong chelating ability. Nam Kim et al. [39] prepared tannic-acid-coated graphene oxide (TA@RGO) by a simple method and used it to prepare polyurethane nanofibers by electrospinning. The cone calorimeter test data related to the modified polyurethane nanofibers show that the pHRR of the PU/5 wt%TA@RGO composite decreased by 30.96% compared with neat PU. In addition, the mechanical properties and antibacterial properties of the modified PU were also improved. Finally, Kim et al. [39] provided some ideas for the preparation of multifunctional composites.

The preparation of natural flame retardants and flame-retardant composites from biomass has become an attractive research topic. Zabihi et al. [40] used fish sperm as a raw material and natural graphite to prepare DNA-functionalized grapheme (GnP^D^) by a ball milling method. The addition of DNA can prevent the aggregation of graphene and play the role of an intumescent flame retardant. The epoxy resin composite with 10 wt% GnP^D^ reached the V-0 level in a UL-94 test.

There are many reports on the preparation of graphene-based flame retardants through various modifications, but the preparation of graphene-based composites with graphene as the matrix remains a rare idea. Hu et al. [41] prepared phosphorus- and nitrogen-modified graphene foams by a freeze-drying-assisted method and a simple heat treatment. The nitrogen and phosphorus mainly come from hexachlorocyclotriphosphate (HCTP). The graphene foams have high flame retardancy, compressibility, and microwave absorption properties and are expected to be applied in the aerospace field.

In recent years, the use of dopamine has gradually become a safe and efficient surface modification method. Luo et al. [42] prepared polydopamine (PDA)-coated reduced graphene oxide thin films based on the reduction and self-polymerization ability of dopamine. The film material has high thermal conductivity and excellent flame retardancy. The peak value heat release rate of the tested films was decreased to near zero according to micro-combustion calorimetry tests.

**Figure 11:**
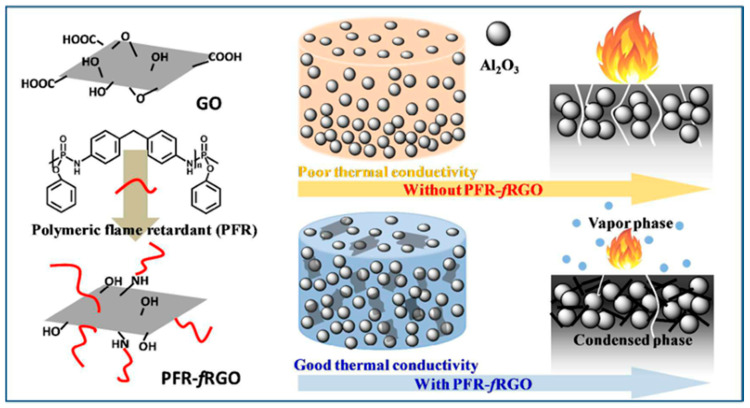
Synergistic enhancement effect of PFR-fRGO and Al_2_O_3_ on the flame resistance and thermal conductivity of epoxy-based composites [43].

Moreover, it is very meaningful to graft many long-chain polymer molecules onto the surface of graphene. Surface-coated grapheme (PFR-fRGO) was prepared by using poly (4,4′-diaminodiphenylmethane phenyl dichlorophosphate). PFR-fRGO can prevent the deposition of alumina, leading to the formation of a three-dimensional heat conduction channel with such alumina. According to Figure 11, the phosphorus and nitrogen elements in the modifier play the role of a flame retardant in the vapor phase and the condensed phase. When the addition amount of PFR-fRGO was 1 wt%, an epoxy resin composite with high thermal conductivity and high flame retardancy was successfully prepared [43].

The phosphorylation of graphene through the polymerization of aniline, pyrrole, and phosphoric acid permits us to improve the dispersion and flame retardancy of graphene. This modification method brings a large number of nitrogen and phosphorus-containing functional groups to the surface of the graphene, which can play the role of an intumescent flame retardant and catalyze the formation of an expanded carbon layer in the combustion process [44].

In addition, chitosan was used as a charring agent to deposit multiple layers on the surface of graphene. Pure polyurethane foam is highly inflammable and produces droplets. Polyurethane foam coated with multiple layers of chitosan-deposited graphene coatings showed excellent flame retardancy. The six pairs of chitosan and graphene layered materials showed excellent flame retardancy due to almost no combustion in the cone calorimetry test [45].

### 2.4. Surface Decoration of Graphene

The surface modification of graphene by various methods is also a very effective process that has attracted the attention of researchers and produced numerous studies. Depending on the different modification methods and modifiers, graphene-based composites with different properties can be obtained. Zinc hydroxystannate boxes (ZHS) are a kind of inorganic flame retardant that acts as a smoke suppressor. Li et al. [46] prepared a new flame retardant called GNS-ZHS-M2070 by covalent grafting of polyether amine (M2070) onto the surface of zinc hydroxystannate box-decorated graphene nanosheets (GNS).The GNS-ZHS-M2070/EP-12% composites show excellent flame retardancy. In addition, zinc-hydroxystannate-modified graphene composites were prepared by the hydrothermal reaction of zinc hydroxystannate and graphene oxide in an autoclave. The pHRR of the GNS-ZHS-M2070/EP-12% composite was obviously lower than that of pure epoxy resin. The combination of zinc hydroxystannate and graphene can significantly reduce the amount of smoke emitted during polymer combustion [47].

Through a variety of components capable of modifying graphene’s surface, it is possible to increase the matrix’s material performance up to a level that complies with different demands mainly related to flame retardancy, mechanical properties, and smoke emissions. For instance, graphene was modified by hexachlorocyclotriphosphate (HCCP) and nickel hydroxide to improve the flame retardant properties in both the gas and condensed phases. In virtue of the low addition amount (3 wt%), the loss of mechanical properties is usually small. Furthermore, some researchers have used polyaniline and nickel hydroxide to perform surface modification in the same line as described above to increase the flame retardant properties. Compared with others, composites with these two flame retardants have better smoke suppression performance, which is mainly due to the excellent flame retardancy of nickel hydroxide and grapheme [48,49].

Another important method for modifying the surface of graphene is based on a hydrothermal process. This method is easy to implement and permits us to prepare phosphorus and nitrogen-modified graphene by the hydrothermal reaction of compounds containing phosphorus and nitrogen. Feng et al. [50] used phosphoric acid and urea as phosphorus and nitrogen sources, respectively. Phosphorus and nitrogen-modified reduced graphene oxide (PN-rGO) was prepared by hydrothermal reaction and micro-blog reduction methods and used in epoxy matrix composites. The peak heat release rate of EP/5 wt% PN-rGO decreased by 30.9%, and this composite reached the V-0 level in the vertical combustion test. In addition, the cited researchers prepared magnesium-phosphate-modified graphene oxide by using magnesium chloride and amino Sanya methyl phosphonic acid as reducing agents. Afterwards, this was applied to a phenolic foam, which not only improved the flame retardancy but also improved the toughness [51].

In addition to the above-mentioned surface modification, ZIF-8 was prepared on the surface of graphene oxide by a hydrothermal method. ZIF-8 can catalyze the formation of carbon, which can further improve the barrier effect of graphene, reducing the amount of smoke released as well. The results show that the epoxy resin composite with 2 wt% ZIF-8/RGO can reach the V-1 level in the vertical combustion test. The composite prepared by adding the same content of ZIF-8 or RGO in epoxy resin cannot reach the V-1 level [52]. Cai et al. [53] modified the surface of graphene by covalent binding of sodium lignosulfonate and iron ions to form flame-retardant-functionalized graphene sheets (FGNS). Due to the high carbonization performance of lignin and the catalytic performance of iron, the flame retardancy of the graphene was further improved. Through adding 2 wt% FGNS, the pHRR of TPU composites decreased by more than half compared with neat TPU.

The environmental risk of this preparation method is relatively high. Attia et al. [54] used diphosphate maleate as a dispersant and a modifier to exfoliate and coat graphene in one step under ultrasonic conditions, forming graphene oxide coated with the remaining diphosphate maleate (GRP). Afterwards, the surface of the exfoliated graphene was modified with titanium dioxide nanosheets with an average size of about 21 nm to form binary modified graphene nanosheets (GRP-MDP-TiO_2_NP). These were added to ABS materials to prepare flame retardant composites. The advantage of this method lies in the simultaneous exfoliation and modification of graphene nanosheets, and it shows excellent performance in inhibiting toxic gas emissions. The peak heat release rate of the ABS/5 wt% GRP-MDP-TIO_2_ NP composite decreased by 49% compared with pure ABS.

Graphene can be directly used as a base material for the modification of a flame retardant. Nie et al. [55] modified a certain amount of graphene oxide on the surface of organic zirconium phosphonate layered materials (GO-Zr(AE)_3_P). This idea is mainly based on the high proportion of oxygen in different functional groups located on the surface of the graphene oxide, which can react with zirconium organophosphate. This process can form a new nano flame retardant. When adding a small amount of GO-Zr(AE)_3_P(≤2 wt%), the peak heat release rate of PP/GO-Zr(AE)_3_P decreased obviously and displayed superior smoke suppression performance. Table 2 summarizes the application of partially modified graphene materials directly as flame retardants in polymer materials mentioned in this paper.

### 2.5. Organic Phosphorus-Containing Flame Retardant Grafted onto Graphene

The preparation of organophosphorus-modified graphene-based flame retardants by the graft reaction of phosphorus-containing compounds and graphene containing oxidation energy groups on its surface has gradually become an attractive research topic [56]. Table 3 summarizes the flame retardant performance of some polymer nanocomposites based on DOPO-modified graphene. DOPO is an intermediate of a phosphorus-containing flame retardant. DOPO contains very active P-H bonds, so it can react with a variety of groups. At the same time, the surface of graphene oxide contains oxygen-containing functional groups, mainly but not limited to hydroxyls and carboxyls. It is common to connect both of them by various bridging agents. Wang et al. [57] modified DOPO with vinyltriethoxysilane and reacted it with graphene oxide modified by isocyanate to prepare a DOPO-VTES-GO flame retardant. Through adding 3 wt% DOPO-VTES-GO, the BDM/DBA composites reached the V-0 level in the vertical combustion test. Qian et al. [58] prepared DOPO-VTS by the reaction of vinyltrimethoxysilane and DOPO. As a surface modifier, DOPO-VTS reacted with oxygen-containing functional groups on the surface of the graphene. Graphene grafted onto the surface of organophosphorus was prepared for the flame retardant modification of polyurea. The peak heat release rate of PUA composites with only 1 wt% functional graphene decreased significantly. In addition to the above methods, DOPO-HQ has also been used to modify the surface of graphene oxide to form DOPO-modified graphene materials (FGO-HQ) with high thermal stability. A PLA/6 wt%FGO-HQ composite reached a V-0 rating in the vertical combustion test [59]. Sun et al. [60] used formaldehyde as a DOPO modifier to prepare DOPO-modified graphene, which was used to achieve a good dispersion of DOPO and graphene in a carbon fiber/epoxy resin (CF/ER) composite. The peak heat release rate of CF/ER/3%graphene–DOPO composites decreased obviously compared with CF/ER composites. Dai et al. [61] modified DOPO with formaldehyde and phosphorus oxychloride, grafted it to graphene, and finally copolymerized it with styrene to obtain a PS-fGO composite. Chen et al. [62] prepared long-chain phosphaphenanthrene (DPP) by a silane coupling agent and used it to modify graphene (DPP-GO). Long-chain compounds were grafted onto the graphene surface to improve the dispersion while introducing an organophosphorus flame retardant. The thermal stability of the composites was improved by the above two modification methods, which displayed excellent performance in LOI, UL-94, and cone calorimetry tests. The epoxy resin composite prepared by adding 3 wt% DPP-GO reached the V-0 level in the vertical combustion test, and the LOI was 25.3%. These results were mainly due to the free radical quenching effect of DOPO during combustion and the physical barrier effect of graphene that was used to form the gas and condensed phases.

**Figure 12:**
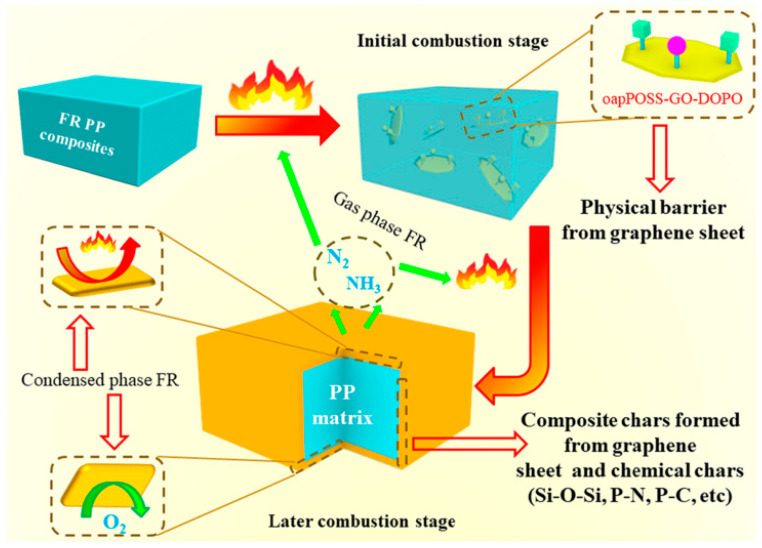
A schematic diagram of the flame retardant mechanism of the PP/P-G-D FR composite [64].

Polyhedral silsesquioxane (POSS) contains a silicon–oxygen bond in its chemical structure. The eight vertices of the POSS cage structure are usually reactive and can be used for grafting and polymerization. POSS itself functions as a flame retardant and a smoke suppressant. POSS-functionalized graphene oxide was prepared by grafting octa (propyl glycidyl ether) POSS (og-POSS) and KH550-modified amino graphene. It is also considered in studies on flame-retardant epoxy resin. The flame retardant properties of EP can be improved obviously by adding 0.7 wt% POSS-functionalized grapheme [63]. Because of the high reactivity of POSS, some researchers have tried to combine organophosphorus flame retardants with POSS and graphene oxide to prepare ternary graft flame retardants. Yuan et al. [64] prepared an oapPOSS-graft-GO-graft-DOPO(P-G-D) flame retardant through the reaction of aminopropylene-modified POSS (oap-p-poss) with DOPO and graphene oxide, and used it as a flame retardant for PP. When the content of P-G-D reached 25 wt%, the PP composites obtained better flame retardancy. Figure 12 shows the flame retardant mechanism of oapPOSS-graft-GO-graft-DOPO. In addition, Zhang et al. [65] polymerized methacryloyloxy butyl POSS, bisdopoma, and glycidyl methacrylate (GMA) as monomers to prepare a flame retardant containing phosphorus, nitrogen, and silicon, which was chemically bonded with graphene oxide to form a hybrid flame retardant (GO-MD-MP). The flame retardant prepared by this method usually contains more than two kinds of flame retardant units and can form a synergistic effect, with the physical barrier property of graphene promoting notable improvements in several properties, including flame retardancy. When 4 wt% go-md-mp is added, the epoxy resin composite can reach the V-0 level in the vertical combustion test.

Similar to DOPO, hexachlorocyclotriphosphate (HCCP) is also a flame-retardant intermediate. It has very active phosphorus–chlorine bonds in its structure and can react with many substances. This property is very beneficial to the surface modification of graphene. Dong et al. [66] realized the combination of graphene oxide and HCCP by an evaporation-induced self-assembly method. The cross-linking effect induced by stacking can form GO-HCCP paper, which can also be used as a flame retardant to add other fibers, leading to acceptable levels of flame retardancy. Cai et al. [67] found a kind of HCCP-modified product that can exfoliate and prepare flame-retardant graphene sheets by an electrochemical method. The modification of thermally conductive epoxy materials with a flame retardant has always been an important topic. The preparation of flame-retardant and thermally conductive epoxy resin by HCCP-modified graphene-oxide-coated alumina microspheres has also been reported [68].

Hu et al. [69] used a self-made hyperbranched phosphorus-containing flame retardant to graft graphene oxide, which brought about a higher nitrogen and phosphorus loading on the graphene. At the same time, due to the steric hindrance effect of the hyperbranched flame retardant, the agglomeration of graphene oxide was prevented. A highly dispersed graphene-based flame retardant was formed in a polystyrene matrix. The peak heat release rate of PS composites with a 2 wt% hyperbranched flame retardant grafted onto the graphene oxide had decreased by 39% compared with neat PS. The flame retardant modification was obtained from the two aspects of the condensed phase and the gas phase, which greatly reduced the amount of smoke emitted from the polystyrene during combustion.

## 3. Carbon Nanotubes

### 3.1. Pristine Carbon Nanotubes

In accordance with carbon nanotubes’ diameter, two categories can be distinguished: single-walled carbon nanotubes and multi-walled carbon nanotubes (MWCNTs). Multi-walled carbon nanotubes are widely used in composites [70]. Carbon nanotubes have excellent mechanical and electrical properties and have been studied in many fields, particularly as a mechanical reinforcing filler for polymer materials. The synergistic effects of CNTs in different flame retardant systems are summarized in Table 4.

Epoxy resin composites filled with thermally oxidized carbon nanotubes were prepared by a casting method, and their flame retardancy was studied by cone calorimetry. In the case of the addition of 1 wt%, compared with the original carbon nanotubes, the thermal-oxidation-treated carbon nanotubes have better flame retardancy properties [71]. The synergistic effect of CNTs on polystyrene electromagnetic shielding material with an intumescent flame retardant was also investigated [72]. The results show that the addition of carbon nanotubes can improve the efficiency of the intumescent flame retardant. Note that when the amount of intumescent flame retardant is greater than 10 wt%, the addition of 1 wt% carbon nanotubes induces better flame retardant performance. The effect of carbon nanotubes on the flame retardancy of thermoplastic polyurethane electromagnetic shielding materials with intumescent flame retardants was also investigated [73]. It was found that the thermoplastic polyurethane composite with 1 wt% carbon nanotubes and 10 wt% intumescent flame retardants had the best flame retardancy. Moreover, the TPU/IFR/CNT system had higher conductivity than TPU with only carbon nanotubes. This may be due to the better dispersion of carbon nanotubes under the friction between and collision of IFR particles and the formation of a conductive network.

The flame retardant modification of a nylon fabric with carbon nanotubes and an intumescent flame retardant has also been reported. The addition of 0.5 wt% carbon nanotubes improved the flame retardancy of the nylon fiber. Compared with the fiber to which only the intumescent flame retardant was added, the heat release rate decreased. This can be explained by the fact that CNTs can improve the quality of the expanded carbon layer, preventing cracks [74]. The synergistic flame retardancy effect of carbon nanotubes and ammonium polyphosphate has also been investigated [75]. The addition of 0.25 wt% CNTs can improve the flame retardancy of APP-modified nylon 6 fiber. However, with the increase in the CNT content, the flame retardancy of nylon 6 fiber gradually becomes worse. This may be due to the excess CNTs that increase the viscosity at melt, which makes the material difficult to foam and reduces the flame retardancy of APP.

Oxidized carbon nanotubes have better dispersion in the matrix. Some researchers have studied the synergistic effect of oxidized carbon nanotubes and a self-made high phosphorus content flame retardant on silicone rubber. When the content of oxidized carbon nanotubes was 1 wt%, the peak heat release rate decreased slightly, but this silicone rubber specimen reached the V-0 level in the vertical combustion test. This was mainly due to the enhancement of the carbon layer by the carbon nanotubes. The mechanical properties of the composites were also improved by adding a small amount of oxidized carbon nanotubes [76].

**Figure 13:**
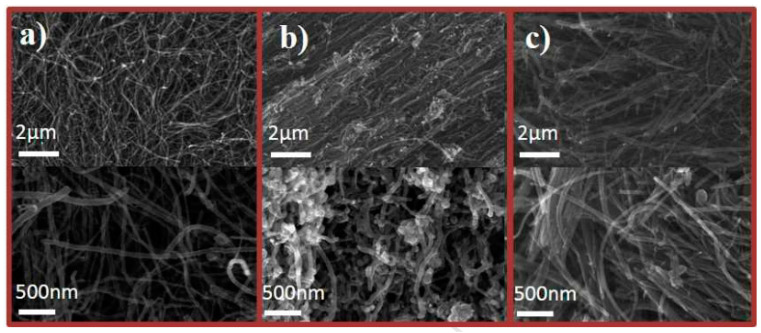
SEM images of: (**a**) CNT, (**b**) COx, and (**c**) CNx at two different magnifications [80].

Chen et al. [77] explored the flame retardant mechanism of carbon nanotubes and montmorillonite nanoparticles in epoxy resin by establishing models that provide a reference for the design of flame retardant formulas and high-performance flame retardants. The flame retardancy of carbon nanotubes and titanium dioxide nanoparticles in PP was also investigated. The addition of titanium dioxide and carbon nanotubes improved the conductivity of polypropylene. It was found that the carbon residue rate of the polypropylene composite with 10 wt% TiO_2_/CNTs was 91.9% after cone calorimetry. This result shows that carbon nanotubes promote the formation of carbon residue [78]. The synergistic flame retardant effect of expandable graphite and carbon nanotubes was also reported. The polyketide was modified by expandable graphite and a small number of carbon nanotubes. When only 1 wt% carbon nanotubes were added, the peak value of the heat release rate decreased significantly compared with polyketide composites with only expandable graphite, which proved the synergistic effect of carbon nanotubes on the flame retardancy of expandable graphite [79]. Novel nitrogen (CNx) and oxygen (COx)-doped CNTs were synthesized by the chemical vapor deposition (CVD) method. From Figure 13, we can see the morphological changes in carbon nanotubes after CVD. Meanwhile, 2 wt% CNx and COx carbon nanotubes were added to epoxy resin to prepare the composite. The epoxy resin modified by COx carbon nanotubes had a limiting oxygen index of 35% and the lowest pHRR. The limiting oxygen index (LOI) of the EP/CNTs composite was only 33.5% at the same addition level. It was proven that the improvement in the flame retardancy of carbon nanotubes doped with oxygen was very significant [80].

**Figure 14:**
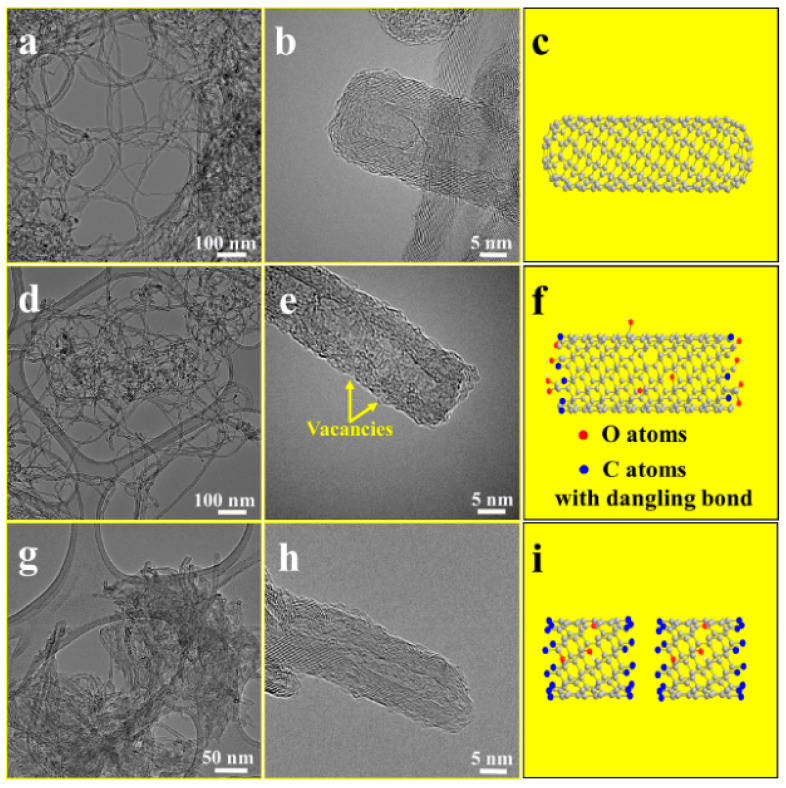
Transmission electron microscopy (TEM) images of the CNTs (**a**,**b**), oCNTs (**d**,**e**), and coCNTs (**g**,**h**); a schematic diagram of a CNT (**c**), an oCNT (**f**), and coCNTs (**i**) [82].

**Figure 15:**
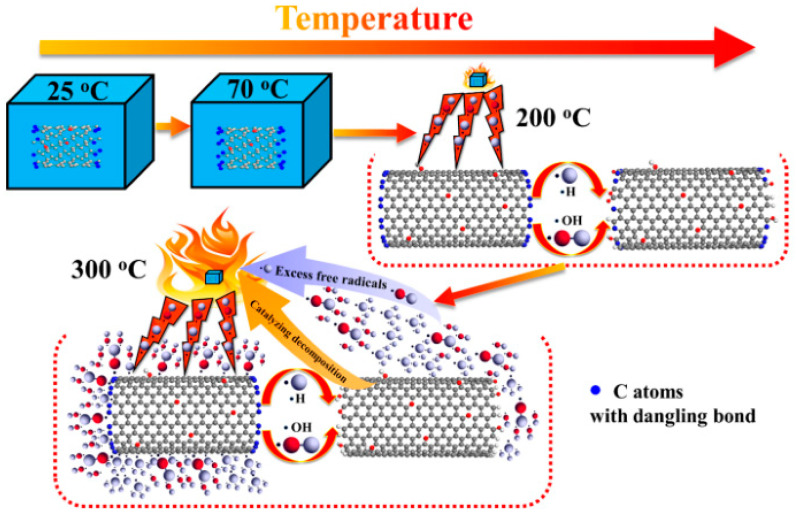
Free radical scavenging effect of the coCNTs during the thermal decomposition of the composite [82].

Research on the preparation of coatings by carbon nanotubes and the preparation of flame-retardant-modified films has also been reported. This is a modification method for the preparation of carbon nanotube films directly without dependence on a polymer matrix [81]. Carbon nanotubes were oxidized by concentrated sulfuric acid, and then the oxidized carbon nanotubes (oCNTs) were spheroidized to prepare carbon nanotubes rich in carbon-centered free radicals (coCNTs) whose flame retardancy and mechanical properties in epoxy matrix materials were investigated. Figure 14 and Figure 15 show the change in morphology of the CNTs after modification and the flame retardant mechanism of coCNTs, respectively. The LOI of epoxy resin composites with 3 wt% coCNTs was 28.4%, while that of pure EP is only 21.9%. The modified CNTs were found to have a stronger ability to capture free radicals and less of an effect on the viscosity at melt. The mechanical properties were also improved [82].

**Table 4:** Synergistic effect of carbon nanotubes on different flame retardant systems.

Matrix	Flame Retardant System	Flame Retardant Performance	Ref
PA66	intumescent fire retardant (IFR)	pHRR and THR were reduced by 76.4% and 76.5%, respectively	[59]
PS	intumescent fire retardant (IFR)	pHRR decreased by 30% and LOI: 34.1%	[72]
TPU	intumescent fire retardant (IFR)	UL-94: V-0 rating, LOI: 30.1%, pHRR and THR were reduced by 92% and 76%, respectively	[73]
PA6	APP	UL-94: V-0 rating, pHRR decreased by more than 30%	[75]
silicone rubber (SR)	DHCP-PA (a high phosphorus content flame retardant system)	UL-94: V-0 rating, LOI: 28.4%	[76]

### 3.2. Surface-Functionalized Carbon Nanotubes

The combination of carbon nanotubes and inorganic flame retardants has also attracted research interest. A zinc–aluminum layered double hydroxide/carbon nanotube hybrid (CNT/ZnAl-LDH) was prepared by the coprecipitation method. Transmission electron microscopy (TEM) showed that the CNTs were embedded between the piles of layered double hydroxides. When the amount of CNT/ZnAl-LDH was 2.1 wt%, the flame retardancy of the PU foam was better. The quality of the carbon residue was improved and the flame retardancy was enhanced by the insertion of carbon nanotubes [83].

The hybridization of organic Fe–Ni layered double hydroxides and carbon nanotubes also yields an effective flame retardant. The dispersion of layered double hydroxides and the carbonization performance of matrix materials can be improved by their hybridization. The results show that the heat release rate and the smoke release rate of composites with only 4 wt% nano fillers had greatly decreased [84]. Multi-walled carbon nanotubes (MWCNTs) were treated with phosphoric acid, and phosphorus was introduced onto the surface of the CNTs to prepare a new type of phosphorus carbon nanotube (P-MWCNT) flame retardant. A polystyrene system was found to have better flame retardancy by compounding 10 wt% P-MWCNTs into the polystyrene. This may be related to the dispersion of carbon nanotubes [85].

A new type of flame retardant was prepared by the polymerization of hexachlorophosphine cyanogen and p-phenylenediamine and coating with the resulting polymer the surface of carbon nanotubes (PCP-CNT), which were used to modify the flame retardancy of PBT. The results show that the samples with 15 wt% PCP-CNT provide optimal properties, which can be attributed to the carbon-forming ability of the P-N polymer and the enhancement of the carbon layer by carbon nanotubes [86].

APP was modified with a silane coupling agent, coated by carbon nanotubes, and named CAPP. It was also used for the flame retardant modification of PBS. The content of CNTs in PBS composites with the best flame retardancy was only 1 wt%. This flame retardant not only has the many advantages of intumescent flame retardants, but also overcomes the disadvantages of reduced mechanical properties and improves the smoke suppression performance. This is mainly due to the formation of a carbon-nanotube-coated APP network, which improves the compatibility of the flame retardant and the matrix. In addition, the quality of the carbon residue was enhanced [87].

**Figure 16:**
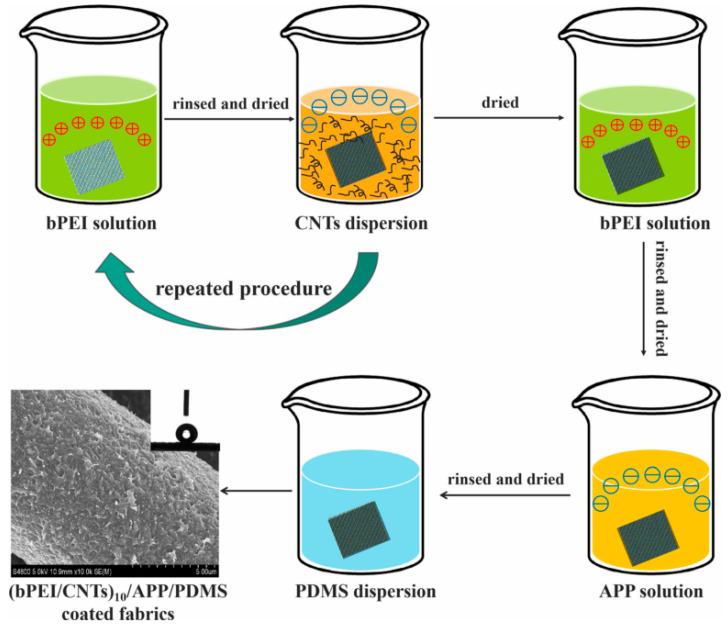
Schematic illustration of the fabrication of multifunctional superhydrophobic fabrics [88].

Flame-retardant cotton fibers were prepared by sequential self-assembly of polyethylenimine, ammonium polyphosphate, and carbon nanotubes on the surface of cotton fibers (Figure 16) [88]. In the vertical combustion test, cotton fibers with 10 wt% bPEI/CNTs showed the characteristics of self-extinguishment. After burning, the cotton fiber showed almost no changes. The fiber also exhibited acid and alkaline resistance, superhydrophilicity, and wear resistance. Carbon nanotubes play an important role in enhancing the carbon layer.

**Figure 17:**
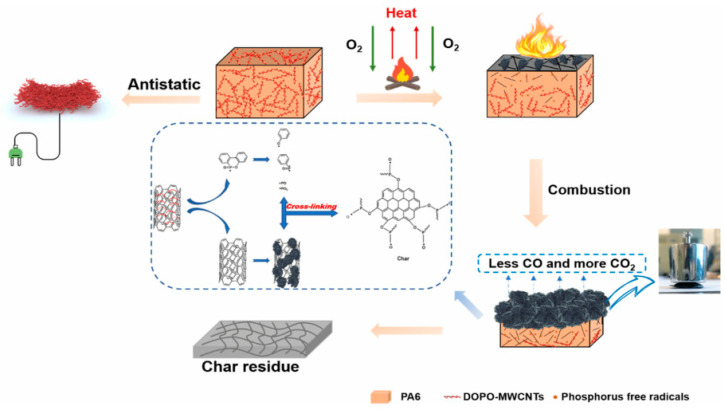
Possible flame retardant and antistatic mechanism of PA6/DOPO multi-walled carbon nanotubes (MWCNTs) [90].

After oxidation, carbon nanotubes form many oxygen-containing functional groups, which are very active and can carry out a variety of reactions. MWCNT-ODOPB prepared by acylation oxidation of carbon nanotubes and reaction with DOPO has been reported to be a flame retardant synergist of aluminum alkyl hypophosphite. By adding 1 wt% MWCNT-ODOPB, the vertical combustion grade of epoxy resin can be improved to V-1. The main function of modified carbon nanotubes is to promote the formation of carbon and improve the strength of the carbon layer [89]. In addition to the above methods, acetaldehyde was used as DOPO modifier to prepare a flame-retardant intermediate, which was then reacted with carbon nanotubes to prepare a composite flame retardant (DOPO-CNTs). The results show that the peak heat release rate of the composites prepared by adding 1.5 wt% DOPO and 1.5 wt% CNTs is 689 ± 10 kW/m^2^. When 3 wt% DOPO-CNTs was added, the peak heat release rate of PA6 composites decreased to 493 ± 10 kW/m^2^. This indicates that the grafting modification is necessary and successful. The possible flame retardant mechanism of DOPO-modified CNTs is briefly summarized in Figure 17 [90].

Other researchers have reported different oxidation states of phosphorus-containing reagents in order to graft amino carbon nanotubes and have explored their role in the flame retardant process of materials. The results show that composites modified by diphenylphosphoryl chloride have the best mechanical properties compared with other PBT specimens, which may be due to the improvement in the interface connection between the carbon nanotubes and the composites. Carbon nanotubes modified by diphenylphosphine chloride improved the flame retardancy of the composites to the greatest extent [91]. A lignin-modified graphene/carbon nanotube hybrid coating was prepared by the Meyer rod method. Paper with a 4 wt% carbon nano hybrid coating was found to have good flame retardancy and a self-extinguishing capability. Lignin can be used as a carbon source, while graphene and carbon nanotubes can enhance the carbon layer and form an effective heat conduction network [92].

**Figure 18:**
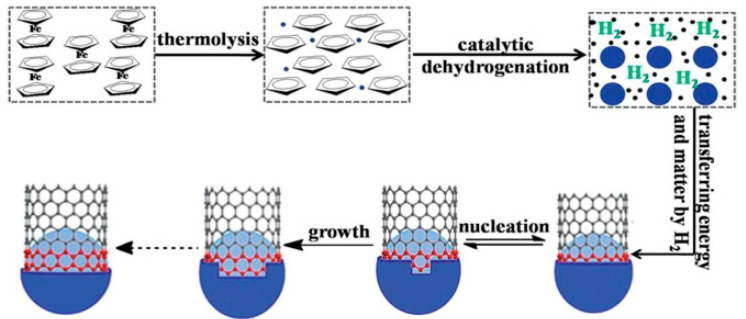
Schematic illustration of the synthetic route to Fe-CNTs on a large scale [94].

**Figure 19:**
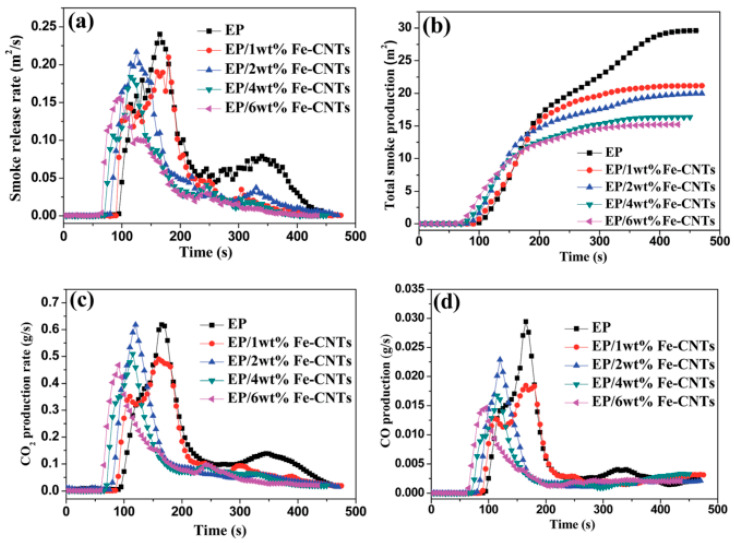
Smoke and gas release of pure EP and EP/Fe-CNT nanocomposites: (**a**) smoke production rate curves; (**b**) total smoke release curves; (**c**) CO2 production curves; (**d**) CO production curves [94].

Some researchers grafted melamine pentaerythritol phosphate (PPMS) onto the surface of carbon nanotubes, which is a one-component intumescent flame retardant. It was used for the flame retardant modification of epoxy resin. Through limiting oxygen index and UL-94 test results, it was found that the addition of 15 wt% PPMS-CNT improved the flame retardancy of the epoxy resin, and the peak heat release rate of the composite was observed to have a very obvious decline in the cone calorimetric test [93]. Zhang et al. [94] prepared a novel type of Fe-CNT on a large scale. The Fe-CNTs were used as a flame retardant in epoxy resin (EP). Figure 18 shows the modification route of Fe-CNTs. Iron carbon nanotubes form a three-dimensional network of well-dispersed carbon nanotubes in the polymer matrix, which can promote the cross-linking of free radicals and the epoxy resin matrix during combustion so as to form a carbon layer with a good structure. The effect of Fe-CNTs on the smoke suppression performance of the epoxy resin was proved by the results of cone calorimetry (Figure 19).

## 4. Fullerene and Other Nanocarbons

Fullerene is an allotrope of carbon. It has a hollow sphere structure, which is usually composed of six-membered rings at a number ranging from five to seven. The free radical trapping effect of fullerenes can improve the flame retardancy of materials and has attracted the attention of many researchers [95]. In addition, other types of carbon nanomaterials, such as expandable graphite and carbon black, have been a source of research as well.

Song et al. [96] prepared a low nitrogen and phosphorus content intumescent flame retardant (PDBPP) and grafted it onto fullerene spheres to prepare a c60-d-PDBPP integrated flame retardant, which was used for the flame retardant modification of PP. The intention was to provide functions related to free radical quenching and an intumescent carbon layer flame retardant. The results show that the flame retardancy and thermal stability of the composites were greatly improved by adding 2 wt% PDBPP.

As pointed out above, fullerenes can capture free radicals. For instance, when aluminum hydroxide decomposes to form an aluminum oxide protective shell, fullerenes capture the free radicals produced by the matrix’s combustion. Adding 60 wt% aluminum hydroxide to SBS can make the composites achieve a V-0 grade in the vertical combustion test. When 3 wt% fullerene and 53 wt% aluminum hydroxide were added, the composite reached the V-0 grade. A good synergistic effect of the flame retardant was formed [97]. Fullerenes are also used in brominated flame retardant systems with high safety. The cited study reports that the addition of bromine and fullerenes has a great influence on the flame retardant performance. HDPE composites with 2 wt% fullerenes were found to have the best flame retardancy. In addition, fullerenes can capture bromine radicals during the combustion process; however, too much fullerene will lead to a decrease in the flame retardancy of a bromine flame retardant [98].

Fullerene may affect the cross-linking of high-temperature vulcanized materials, such as silicone rubber, resulting in a decline in the material’s performance. A modified fullerene was prepared by the combination of polymethylphenylsiloxane and fullerene by the stacking effect (PMPS-d-C60). By adding 4 phr pmps-d-c60 to the silicone rubber, the LOI reached 29.0%, showing excellent flame retardancy [99]. The combination of DOPO and fullerene is also an important flame retardant modification method. Following this approach, it is possible to enhance the free radical capture ability of fullerenes and prolong the ignition time. By adding 3 wt% DOPO-d-C_60_, the peak of the heat release rate of PP composites was decreased slightly [100].

**Figure 20:**
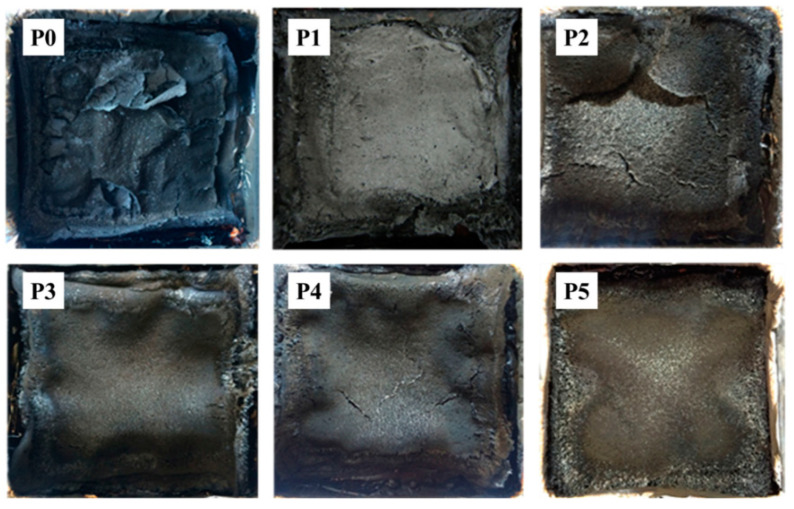
Digital photos of carbon residue after the burning of the RPUF samples [102]. P0: pure RPUF; P1: 20phppDPES/RPUFs; P2: 5phppIL-EG/15phppDPES/RPUFs; P3: 10phppIL-EG/10phppDPES/RPUFs; P4: 15phppIL-EG/5phppDPES/RPUFs; P5: 20phppIL-EG/RPUFs (phpp: per hundred of polyether polyol by weight.).

Expandable graphite (EG) is a low-cost, one-component intumescent flame retardant and also a kind of nano carbon material. When the content of expandable graphite, huntite, and hydromagnesite is 25 wt%, the TPU composite reaches the V-0 level in the vertical combustion test and the highest limiting oxygen index. With the increase in the expandable graphite content, the mechanical properties of TPU composites are improved, which overcomes the disadvantage of the mechanical properties of TPU composites due to the addition of the inorganic flame retardant [101]. Expandable graphite modified by an ionic liquid containing phosphorus and a phosphorus and nitrogen intumescent flame retardant were synthesized to form a synergistic flame retardant system named IL-EG/DPES. In addition, they were used for the flame retardant modification of rigid polyurethane foams (RPUFs). When the content of both IL-EG and DPES was 10 phpp, RPUFs had the best flame retardancy. Due to the simultaneous effect of the intumescent flame retardant and the ionic-liquid-modified expandable graphite, a strong expanded carbon layer was formed. By observing the carbon residue after cone calorimetry, a reinforcing effect of the modified expandable graphite on the carbon layer was found (Figure 20). The results also show that the total amount of smoke emitted decreased significantly [102].

Yun et al. [103] studied the synergistic effect of expandable graphite and phosphorus-based flame retardants. They introduced iron phosphate coordination network to the surface of microcrystalline cellulose through PDA. It is used as flame retardant synergistic agent for expandable graphite/polyurethane foam composite. The appearance of an expanded carbon layer can protect the matrix material.

Carbon black (CB) is also an important nano carbon material. In most cases, carbon black appears as a reinforcing modifier of rubber. However, carbon black can be used as a synergist to improve the flame retardant efficiency of other flame retardant materials, especially magnesium hydroxide. The addition of 0.5 wt% carbon black can make EVA/MH composites reach the V-0 level in the vertical combustion test and improve the mechanical properties of the matrix material to a certain extent [104]. It is also very meaningful to graft carbon black. When the one-component intumescent flame retardant named pentaerythritol phosphate melamine salt (PPMS) was grafted onto carbon black and added to epoxy resin as a flame retardant and smoke suppressant, the EP/15 wt% PPMS-CB composite had excellent performance in cone calorimetry, and the peak heat release rate and smoke release were greatly reduced [105].

**Figure 21:**
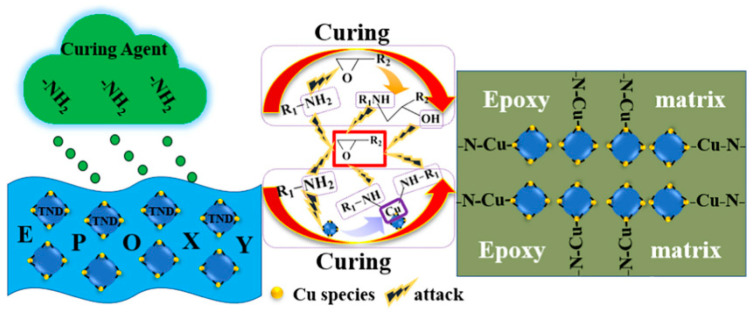
Proposed mechanism for the reinforcing effect by TND [106].

Nanodiamond (ND) was modified with P species, Cu species, and (2,2,6,6-tetramethylpiperidin-1-yl)oxyl (TEMPO) step by step in order to prepare a flame retardant and strengthen the mechanical properties of nano carbon materials for epoxy composites. Nanodiamond was used as a carrier to connect copper, tempo resin, and epoxy resin. The mechanical properties and flame retardancy of the composites were further enhanced by forming an interconnected network with the epoxy resin matrix during the curing process. Figure 21 shows that the coupling agent on the surface of the nanodiamond participates in the curing process of epoxy resin. The nanodiamond can form a reinforcing network in the epoxy resin matrix [106].

**Figure 22:**
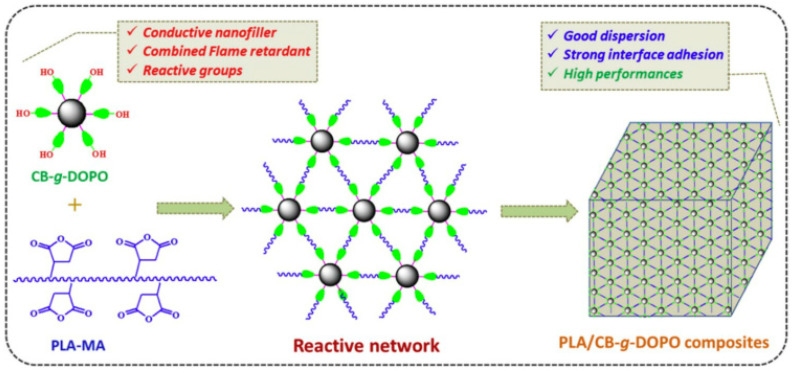
Schematic illustration of the chemical reaction between CB-g-DOPO and PLA-MA to form a reactive network in the PLA matrix [107].

DOPO (9,10-dihydro-9-oxy-10-phosphazene-10-oxide) is an effective phosphorus-containing flame retardant. However, DOPO easily migrates to the polymer matrix material, which reduces the flame retardant’s efficiency. By introducing carbon black as the basis of a modification, DOPO was grafted onto the surface of hydroxylated carbon black to prepare hybrid materials (CB-g-DOPO) with a high grafting rate. The PLA/8 wt% CB-g-DOPO composite not only has strong flame retardancy but also shows a certain degree of conductivity. As shown in Figure 22, the CB-g-DOPO reacted with maleic anhydride in PLA-MA. This reaction realizes the combination of the modifier and the matrix, hinders the diffusion of the flame retardant, and improves the dispersion of the flame retardant [107].

**Figure 23:**
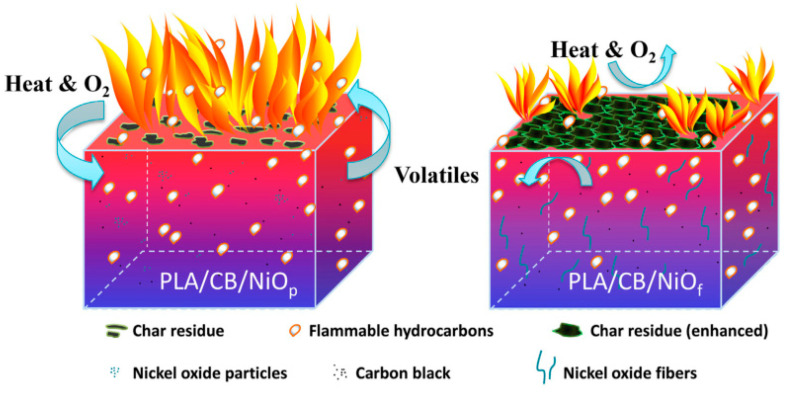
Proposed flame retardant mechanism for PLA composites with carbon black and NiO particles/fibers [108].

Zhang et al. [108] fabricated porous submicron nickel oxide (NiO) fibers via an electrospinning and subsequent pyrolysis process. Carbon black (CB) can also be combined with NiO to form a reticulated flame retardant system and plays the part of a connection point in this system. The presence of carbon black enhances the carbonization ability of NiO. CB/NiO_f_ mainly plays the role of a condensed-phase flame retardant in PLA’s combustion (Figure 23).

MXene can not only be combined with other nano carbon materials to prepare flame retardant materials, but also has the potential to become a flame retardant. However, MXene is easily oxidized in air and easily agglomerates when applied to polymer materials. Ning et al. [109] deposited nano silica onto the surface of MXene by in situ hydrolysis of tetraethyl orthosilicate. Then, functional MXene (m-MXene) was prepared by a silane coupling agent modification. Flame-retardant composites were prepared by compounding with polyvinyl alcohol. The PHRR of m-MXene/PVA composites prepared with only 2 wt% m-MXene was reduced by 34.9% and 16.9%, respectively, compared with pure PVA and PVA composites prepared with 2 wt% unmodified MXene. These results show that surface modification can improve the dispersion of MXene in the matrix material and improve the flame retardant performance. In addition, the mechanical properties of m-MXene/PVA composites were also improved. Compared with pure PVA, the tensile strength and elongation at break of the composites were increased by 32.9% and 97%, respectively. Because MXene itself has electrical conductivity, the electrical conductivity of the composites was also improved. The surface modification of MXene is very important to the improvement of flame retardants and the mechanical properties of composites. Through surface modification, the dispersion can be improved and the interfacial compatibility between additives and matrix materials can be improved.

## 5. Summary and Perspective

In recent years, nano carbon-based materials have been widely used to modify polymers with flame retardants. Compared with traditional flame retardants, nano carbon-based materials can significantly improve the flame retardancy, mechanical properties, and thermal and electrical conductivities of multi-functional flame-retardant nanocomposites. Most carbon nanomaterials have special shapes, which are closely related to their excellent properties. Moreover, nano carbon-based materials can be used in a variety of matrix materials or directly made into single-component materials with certain physical properties. Some researchers [81] used carbon nanotubes as the matrix to prepare paper, which provides a new idea for the preparation of novel nanocomposites.

The mechanism of nano carbon materials as flame retardants in different polymer matrices is similar to that of traditional flame retardants. There are two main ways to delay the combustion of the polymer matrix:

(I) The addition of nano carbon material, which helps to form a dense protective carbon layer. Adding nano carbon materials leads to a more compact carbon layer formed by combustion (with fewer cracks and holes). These carbon layers can protect the polymer material at the bottom and block the combustible gas generated so as to protect the matrix material.

(II) Some nano carbon materials, such as fullerenes, have the ability to capture free radicals. They absorb the active free radicals produced during combustion and terminate the chain reaction.

Due to their limited function as a flame retardant, in most cases pristine nano carbon materials are used as synergists together with traditional flame retardants. Moreover, obtaining a good dispersion of pristine carbon nanomaterials in the polymer matrix remains challenging. A poor dispersion of carbon nanomaterials may lead to a deterioration of the mechanical properties. In order to avoid these shortcomings, the surface modification of carbon nanomaterials can be performed. There are two goals in this modification: (i) to reduce the interaction between the carbon nanomaterials and improve their dispersion in the polymer matrix; and (ii) to introduce functional groups in the molecular chains with flame retardancy.

Although some nano carbon materials have excellent properties, the development of low-cost preparation methods for most of the carbon nanomaterial remains challenging. These facts make nano carbon materials difficult to apply on a large scale. We expect that more and more researchers will contribute to low-cost production methods for nano carbon materials. Moreover, the surface functionalization of carbon nanomaterials is quite important to flame retardant applications and will be one of the main research topics in the future. In addition, to date, few researchers have focused on the recyclability of carbon nanocomposites. With the increasing awareness of environmental protection and energy conservation, it would be very important to improve the recyclability of materials and, in particular, develop a new strategy for recycling thermoset-based carbon nanocomposites. An interesting approach would be the use of waste thermoset-based carbon nanocomposites to prepare high-value-added carbon materials.

## Figures and Tables

**Table 2:** Application of modified graphene nanomaterials as flame retardants in polymer composites.

Polymer	Loading of Modified Graphene Nanomaterials	Flame Retardant Performance	Ref
EP	graphenite-Cu3 wt%	LOI value: 26.4%, PHRR decreased from 1193 kW/m^2^ (neat EP) to 786 kW/m^2^	[24]
EP	2 wt% rGO@LDH	65.9% and 16.7% reduction in PHRR and SPR, respectively	[31]
EP	2 wt% GO@MCM-41	40.0% reduction in the peak heat release rate (pHRR)	[34]
PU	5 wt% PDA/GO coating	65% reduction in the peak heat release rate (pHRR) compared with neat PU	[36]
EP	5 wt% PN-rGO	30.9% and 29.3% reduction in PHRR and THR, respectively	[50]
ABS	5 wt%GRP-MDP-TIO2NP	PHRR and THR both dropped by 49%.	[54]

**Table 3:** DOPO-modified graphene for different matrix materials.

Matrix	Types of Grafting Reaction	Main Performance	Ref.
4,4-bismaleimidophenylmethane/2, 2-diallyl bisphenol A (BDM/DBA) resins	Vinyl trie-thoxy silane and (3-isocyanatopropyl)-triethoxysilane as bridging agents	UL-94: V-0 rating, LOI: 32.8%	[57]
PUA	vinyltrimethoxy silane and (3-Isocyanatopropyl)-triethoxysilane as bridging agents	49% reduction in PHRR observed in cone calorimetry	[58]
PLA	Reaction of 2,5-dihydroxyphenol with an acyl chloride bond	83% reduction in TSR observed in cone calorimetry compared with neat PLA	[59]
CF/EP	Reaction of formaldehyde-modified DOPO with an acyl chloride bond	PHRR of composites reduced by 38.9% compared with neat CF/EP	[60]
PS	Paraformaldehyde, hydroxyethyl acrylate, and POCl_3_ as bridging agents	39.1% reduction in PHRR observed in cone calorimetry	[61]

## Abbreviations

***polymers and other compounds***	POSS, Polyhedral silsesquioxane;
MoS2,molybdenum disulfide;	GMA, glycidyl methacrylate;
EVA, ethylene vinyl acetate;	BDM/DBA, 4,4-bismaleimidophenylmethane/2, 2-diallyl bi-sphenol A resins;
MMT, Montmorillonite;	PUA, Polyurethane acrylate;
GO, Graphene oxide;	PS, polystyrene;
LDH, layered double hydroxide;	PK, polyketone;
DOPO, 9,10-dihydro-9-oxa-10-phosphaphenan-threne-10-oxide;	CNTs, carbon nanotubes;
EP, epoxy resin;	PA66, Polyamide66;
PLA, Polylactide;	SR, silicone rubber;
α-ZrP, α-zirconium phosphate;	PPMS, melamine pentaerythritol phosphate;
CPO, cerium phosphate;	IFRs, intumescent flame retardants,
APP, Ammonium polyphosphate;	MWCNT: multi-walled carbon nanotube;
BPPT, Bio-based polyphosphate;	EG, Expandable graphite;
IG, iron–graphene;	CB, Carbon black;
TPU, thermoplastic polyurethane;	ND, Nanodiamond
MH, magnesium hydroxide;	***parameters and characterization***
PBT, polybutylene terephthalate;	Tig, ignition temperature;
AHP, aluminum hypophosphite;	HRR, heat release rate;
BP, Black phosphorene;	PHRR, peak heat release rate;
MZF, Mesoporous zinc ferrate;	MLR, mass loss rate;
MOF, metal–organic framework;	TSP, total smoke product;
AH, Aluminum hydroxide;	THR, total heat release;
RP, Red phosphorus;	EHC, effective heat of combustion;
LBL, layer-by-layer;	FGI, fire growth index;
TA, Tannic acid;	D, thermal diffusivity;
PDA, polydopamine;	SEM, scanning electron microscope;
PFR, polymeric flame retardant;	CCT, Cone calorimeter test;
ZH, Zinc hydroxystannate	H-TRIS, heat-transfer rate inducing system;
HCCP, hexachlorocyclotriphosphate;	TGA, thermogravimetric analysis;
ABS, acrylonitrile–butadiene–styrene;	LOI, limit oxygen index;
DPP, phosphaphenanthrene;	TEM, transmission electron microscope
